# From childhood to adolescence: Development of binge eating and the prospective role of self-regulation

**DOI:** 10.1186/s40337-025-01330-x

**Published:** 2025-07-06

**Authors:** Nele Westermann, Annette M. Klein, Robert Busching, Petra Warschburger

**Affiliations:** 1 Department of Psychology, University of Potsdam, Karl-Liebknecht-Straße 24/25, 14476 Potsdam, Germany; 2 International Psychoanalytic University Berlin, Stromstr. 1, 10555 Berlin, Germany

**Keywords:** Binge eating, Self-regulation, Middle childhood, Adolescence, Prospective

## Abstract

**Background:**

Research shows that binge eating often starts in childhood or adolescence, but its development remains largely unexplored. Additionally, while cross-sectional studies link self-regulation to binge eating, longitudinal research is lacking. Therefore, this study examined the development of binge eating and self-regulation as a potential predictor for this development in a community sample.

**Methods:**

A total of *N* = 1660 children were assessed at four time points spanning ages 6–11, 7–11, 9–13, and 16–21. The assessment of self-regulation encompassed emotional reactivity, working memory updating, cognitive flexibility, inhibition, inhibitory control, planning behavior, affective decision-making, anger regulation, and as appetite self-regulation, satiety responsiveness, emotional overeating, food responsiveness, and external eating, using computerized tasks, teacher- and parent-reports. Binge eating was modeled by child-reported loss of control eating, overeating, and eating in the absence of hunger. A latent change score model was used to evaluate intra- and interindividual differences in binge eating across middle childhood and adolescence. Self-regulation facets were regressed on changes in binge eating.

**Results:**

Results indicated a decrease in binge eating at the beginning of middle childhood, followed by a stagnation and then an increase during adolescence, with significant interindividual differences in these changes. Higher planning behavior, inhibitory control, and cognitive flexibility predicted decreases in binge eating during middle childhood, while higher satiety responsiveness unexpectedly predicted an increase in binge eating during adolescence. Results remained the same after controlling for body weight.

**Conclusions:**

Our findings highlight adolescence as a critical period for binge eating prevention, with planning behavior, inhibitory control, and cognitive flexibility acting as protective factors in middle childhood. The longitudinal data underscore the importance of self-regulation in the development of binge eating.

**Supplementary Information:**

The online version contains supplementary material available at 10.1186/s40337-025-01330-x.

## Abstract (plain language summary)

Binge eating refers to eating large amounts of food while feeling unable to stop. Research shows it can start in childhood, but we still do not fully understand how it develops over time and what influences this process. One important factor might be self-regulation– the ability to manage emotions, thoughts and behavior. In this study 1660 children were followed at four time points from middle childhood to adolescence. Different self-regulation skills were measured with tasks, parent and teacher reports. Binge eating was assessed through child reports. Results indicated that binge eating first decreased in middle childhood, stayed stable for a while, and then increased during adolescence. However, these changes were different for each child. Children with better planning skills, more flexible thinking and a better ability to hold back automatic reactions were less likely to develop binge eating. Surprisingly, children who were more sensitive to feeling full were more likely to binge eat during adolescence. This study shows that good self-regulation skills may protect children from developing binge eating.

**Supplementary Information:**

The online version contains supplementary material available at 10.1186/s40337-025-01330-x.

## Theoretical background

Binge eating is characterized by consuming a large amount of food within a limited period of time accompanied by a sense of loss of control over one’s eating [1]. When binge eating occurs regularly (at least once a week over the last three months), it is one of the core symptoms of eating disorders like bulimia nervosa or binge eating disorder [2]. Binge eating can therefore be viewed as a continuum of increasingly uncontrolled eating behaviors, with the clinically relevant eating disorders at the end of this spectrum [3, 4]. The onset of these disorders typically occurs in adolescence and emerging adulthood [5], but recent studies stress its initial manifestation already earlier in life with an estimated lifetime prevalence of 1–3% for binge eating disorder among children and adolescents [6]. Regarding binge eating, self-report data from German community samples of children and adolescents indicated prevalence rates of 23% for binge eating within the last month [7, 8]. An increase in binge eating has been particularly observed during adolescence, which can be discussed from a developmental perspective with the onset of puberty [9] or as a result of increasingly independent decisions e.g., regarding food choices and eating habits [10]. However, there is currently little information on the exact development and course of binge eating in childhood and adolescence [11].

Understanding the progression of binge eating during childhood and adolescence is essential, given its harmful consequences, including an increased likelihood of requiring eating disorder treatment, experiencing higher rates of victimization, and engaging in substance abuse [12]. Additionally, binge eating is associated with the development of overweight and obesity [13, 14]. Given the high prevalence of binge eating during childhood and adolescence and its far-reaching associations with social, psychological, and physiological life outcomes, it is essential to further understand putative risk and protective factors during development.

Self-regulation plays an important role in the emergence and maintenance of eating disorders and eating behaviors [15]. Self-regulation is defined as the ability to control emotions, behavior, and cognition in order to reach future goals and benefits [16–18]. Self-regulation can be viewed as a multifaceted construct, consisting of both basal and complex processes [18, 19]. Basal self-regulation refers to automatic and effortless mechanisms like cognitive flexibility, working memory updating, inhibition, and emotional reactivity [19]. Complex processes are assumed to build on these basal facets and refer to more effortful, conscious, and cognitively demanding processes like planning behavior, emotional decision-making, and emotion regulation [20]. In recent discussions, appetite-related self-regulation such as emotional overeating, satiety responsiveness, external eating, and food responsiveness are proposed to be part of the complex self-regulation mechanisms [21]. Self-regulation is associated with a wide range of outcomes during childhood and adolescence [15], including individual outcomes such as academic success, weight status [22], and internalizing symptoms [23], as well as social factors such as peer rejection and victimization [24]. Additionally, self-regulation plays a key role in everyday eating behavior for a review see [25], highlighting the importance of examining its potential influence on binge eating.

Indeed, neuropsychological studies have shown that lower self-regulation can contribute to binge eating [1]. Theoretically, this association can be explained by binge eating being a reaction to complex situations and stress, where adaptive processing of the experience would require efficient self-regulation [26]. The association between specific facets of self-regulation and binge eating has thus far been investigated primarily in adults with clinical binge eating disorder, using cross-sectional data. Several meta-analyses and reviews indicated negative associations between binge eating disorder and working memory, emotion regulation, and planning behavior [27–29]. Results regarding inhibition have been inconsistent [27, 30]. Studies involving community samples, as well as children and adolescents, are relatively scarce. Ramalho et al. [31] reported in their systematic review a link between inhibitory control/ inhibition difficulties and loss of control eating in adolescent community samples. However, they report that their results are limited due to the small number and high heterogeneity of studies.

While associations between self-regulation and binge eating have been identified for individual facets, a comprehensive, simultaneous examination of multiple self-regulation facets is lacking. Furthermore, as these results stem from cross-sectional research, it is not yet possible to determine the direction of these associations [32]. While lower self-regulation might lead to higher binge eating, the opposite direction might also be possible. In a prospective study, Schaumberg et al. [33] found that better inhibition, but not working memory, at the age of 10 was associated with a lower risk of disordered eating four years later. The only other prospective study we are aware of found that binge eating at the age of 9–13 years predicted lower planning behavior two years later, which in turn predicted higher binge eating behavior at the age of 14–17 years [34].

### The current study

In summary, there is a lack of prospective community-based studies investigating the development of binge eating in childhood and adolescence. Additionally, a deeper understanding of the risk factors for the development of binge eating in childhood and adolescence is crucial for developing effective prevention and treatment interventions [35]. Therefore, this study aims to better understand deficient self-regulation as a potential risk factor for developing binge eating to identify children and adolescents at risk. It was expected that an increase in binge eating over the course of development would be predicted by lower self-regulation abilities namely higher emotional reactivity, lower working memory updating, lower cognitive flexibility, lower inhibition, lower inhibitory control, lower planning behavior, lower affective decision-making, lower anger regulation, and lower appetite self-regulation (lower satiety responsiveness, higher emotional overeating, higher food responsiveness, higher external eating). Furthermore, this study aimed to explore particularly relevant facets by simultaneously examining a wide range of basal, complex, and appetite-related facets. Additionally, given the expected peak increase in binge eating during adolescence, the strongest predictions were anticipated from self-regulation at the end of middle childhood on the development of binge eating during adolescence. Moreover, sex differences were explored.

## Method

### Procedure

Data were collected as part of the longitudinal community-based PIER study investigating intrapersonal developmental risk factors in childhood and adolescence; for further details, see study protocol [19]. Participants provided information at up to four time points between 2012 and 2024 with a mean interval of approximately nine months between T1 and T2 (*M* = 273.3 days, *SD* = 55.5), 24 months between T2 and T3 (*M* = 23.5 months, *SD* = 1.67), and eight years between T3 and T4 (*M* = 7.93 years, *SD* = 0.49). Recruitment took place in 33 primary schools in urban and rural areas of the federal state of Brandenburg, Germany. From T1 to T3 the participating children were asked to complete a standardized test battery in two separate sessions lasting a total of two hours at each time point. Additionally, parents and teachers completed questionnaires. The children received small gifts for participation. At T4, data collection was performed with a two-hour online session followed by an up to 30-minute face-to-face onsite session. Parents were again asked to fill in questionnaires. Each adolescent received 30 € after participating in both sessions.

For T1 - T3 informed written consent was obtained from each of the child’s primary caregivers and the participating schools, as well as assent from each child. At T4, each participant provided written informed consent and if the participant was under 18 years of age, written consent was additionally obtained from the primary caregiver. The study received approval from the local Research Ethics Committee and the Ministry of Education, Youth, and Sport of the Federal State of Brandenburg. To enhance transparency, the study’s hypotheses and analysis plan were preregistered at https://osf.io/jaq4h. Deviations from preregistration are tracked in Additional file 1, Table S5.

### Participants

At T1, data was available for *N* = 1657 children aged 6–11 (*M* = 8.36 years, *SD* = 0.95 years, 52.2% female), encompassing 1651 child-reports, 1340 parent reports, and 1424 teacher reports. At T2, *N* = 1612 children aged 7–11 (*M* = 9.11 years, *SD* = 0.93 years, 51.9% female) participated again, encompassing 1609 child-reports, 1197 parent-reports, and 1175 teacher-reports. At T3, data was available for *N* = 1534 children aged 9–13 (*M* = 11.06 years, *SD* = 0.92 years, 51.5% female), encompassing 1505 child-reports, 1070 parent-reports, and 1113 teacher-reports. Finally, at T4, data was available for *N* = 613 adolescents aged 16–21 (*M* = 18.92 years, *SD* = 1.04 years, 54.4% female, 2.1% divers), encompassing 570 self-reports and 457 parent-reports. Since data for three children were only available from T2 onwards, the total sample consisted of *N* = 1660.

At T1, the highest reported educational level of the parents was a university degree for 36.6%, a high school diploma (“Abitur”) for 15.5%, higher secondary school for 25.5%, lower secondary school for 2.1% and special school or no degree for 0.9%. 19.6% did not provide information on their educational level. Regarding the weight status at T1, 5.8% of children were underweight, 80.3% normal weight, 7.6% overweight, 5.2% obese, and 1% had height or weight not measured.

### Measures

#### Binge eating

Binge eating at T1-T4 was measured by child-report using the control item of the SCOFF questionnaire [36], which assesses loss of control eating (“Do you worry that you have lost control over how much you eat?”) and two items adapted from the Questionnaire of Eating and Weight Patterns [37] that assess overeating (“Do you eat until you’re uncomfortably full?”), and eating in the absence of hunger (“Do you eat really big amounts of food even when you are not hungry?”). All items were rated on a dichotomous scale (no (0), yes (1)). The three indicators were used for latent modeling at the four time points. Higher values indicate more binge eating.

#### Working memory updating

The ability to monitor and manipulate task-relevant information in the working memory [38] was assessed at T1-T3 by the Digit Span Backwards Task [39]. Participants were asked to verbally repeat number sequences in reverse order. Each of eight trials comprised two sequences of identical length, increasing by one per trial. The test ended after eight trials or two incorrect sequences in a trial. The total number of correctly completed sequences determined the updating score, with higher scores indicating higher updating abilities.

#### Cognitive flexibility

A child-appropriate computerized cognitive flexibility task assessed the ability to flexibly shift between tasks and mental sets at T1 and T2 [40]. Children were instructed to feed one plain and one multi-colored fish alternately across 46 trials, including 22 switch trials in which the location of the fish changed, requiring an adaptation of the response pattern. At T3, an adapted, computer-based version of the Dimensional Change Card Sorting task was used [41]. Children saw two geometrical symbols differing in shape and color and sorted a third colored symbol according to one of two dimensions as quickly and accurately as possible. While the sorting dimension remained constant for the first 20 trials, it changed in 12 out of 48 trials in the second block (switch trials). Anticipation errors (reaction time < 200 ms) and omission errors (three standard deviations above the mean) were excluded. The number of correct responses in switch trials determined the cognitive flexibility score, with higher scores indicating higher cognitive flexibility.

#### Inhibition

The ability to suppress dominant impulses in favor of goal-oriented responses [38] was measured at T1-T3 through an adapted version of the fruit–vegetable Stroop task [42]. The task consisted of four trials à 25 stimuli: Trial 1 encompassed colored rectangles, trial 2 correctly colored fruits, trial 3 fruits colored in grey, and trial 4 incorrectly colored fruits. The children were instructed to name the stimuli color (trial 1 and 2), or the appropriate fruit color (trial 3 and 4). An interference score was calculated [43] and inverted so that higher scores indicate less interference and therefore higher inhibition.

#### Affective decision-making

The tendency to make decisions driven by emotions and to take risks was assessed at T1-T3 by an age-appropriate version of the Iowa Gambling Task called the Hungry Donkey Task [44]. Children collected as many apples as possible across 10 practice and 50 test trials by selecting one of four doors. After selection, gains and losses were presented. Unknown to the participants, two doors were disadvantageous with higher immediate gains but resulting in long-term losses, and two doors were advantageous with lower immediate gains but long-term gains. A higher net score (advantageous minus disadvantageous choices) reflects a preference for advantageous doors and therefore better decision-making.

#### Emotional reactivity

Intensity, speed, and duration of emotional responses were measured at T1-T3 through parent reports using the subscale emotion control of the Behavior Rating Inventory of Executive Function BRIEF; [45]. Parents rated the ten items on a modified 5-point scale ranging from *never* (1) to *always* (5). A mean score was calculated with higher scores indicating higher emotional reactivity and therefore lower self-regulation. The scale indicated an ordinal alpha of α_T1_ = 0.93, α_T2_ = 0.93, and α_T3_ = 0.94.

#### Planning behavior

The ability to master current or future tasks was assessed at T1-T3 through teacher reports using the subscale planning and organizing of the BRIEF [45]. Teachers rated eight items on a modified 5-point scale ranging from *never* (1) to *always* (5). Items were reverse-coded, and a mean score was calculated so that higher means indicate better planning abilities. The items indicated an ordinal alpha of α_T1_ = 0.95, α_T2_ = 0.95, and α_T3_ = 0.96.

#### Inhibitory control

The ability to suppress inappropriate responses in new or uncertain situations was measured at T1-T3 through parent reports using the subscale inhibitory control of the Temperament in Middle Childhood Questionnaire [46]. Parents rated six items on a five-point scale ranging from *not true* (1) to *true* (5). A mean score was calculated, with higher scores indicating higher inhibitory control. The items indicated an ordinal alpha of α_T1_ = 0.73, α_T2_ = 0.72, and α_T3_ = 0.76.

#### Anger regulation

The ability to manage and change anger was measured at T1-T3 through parent-reports using the German Questionnaire on Emotion Regulation in Children and Adolescents FEEL-KJ [47]. At T1 and T2, parents rated four items assessing the anger regulation strategies preservation (1 item), distraction (1 item), and venting (2 items) on a 3-point scale ranging from *never* (1) to *always* (3). At T3, one item per strategy was used. Items assessing venting and perseveration were recoded [48], and a mean score was calculated, with higher mean scores indicating better anger regulation. The items indicated an ordinal alpha of α_T1_ = 0.62, α_T2_ = 0.56, and α_T3_ = 0.43.

#### Appetite self-regulation

The ability to respond to internal cues of hunger and satiety was assessed at T1-T3 through parent-reports using the Children’s Eating Behavior Questionnaire [49] for the facets of *emotional overeating*, *satiety responsiveness*, and *food responsiveness*. For each facet the original scale was shortened to the three items with the highest factor loadings in a pilot test. Items were rated on a 5-point scale from *never* (1) to *always* (5). The Dutch Eating Behavior Questionnaire [50] was used to assess the facet *external eating*. The five items were rated on a 4-point scale *never* (1) to *often* (4). Mean scores were calculated per facet. Higher emotional overeating, food responsiveness, and external eating indicate lower appetite self-regulation, whereas higher satiety responsiveness indicates higher appetite self-regulation. Ordinal alpha ranged between α = 0.75 and α = 0.94.

#### Control variables

Children reported their sex as female or male. The child’s birth date was assessed, and age was calculated. Additionally, children’s body height and weight were measured using standardized equipment and procedures. A standard deviation score of the body mass index (BMI-SDS) was calculated based on sex- and age-specific national reference data [51]. These BMI-SDS scores were used as continuous variables in all main analyses. Weight groups were classified (< 10th percentile underweight; > 90th percentile overweight; >97th percentile obese; > 99.5th percentile severely obese) and served descriptive purposes.

### Data analysis

Structural equation modeling was performed using M*plus* Version 8.11. Binge eating was modeled as a latent variable at each time point. Given the binary nature of the binge eating indicators, the Weighted Least Squares Mean and Variance-adjusted (WLSMV) estimator was used. The WLSMV estimator is especially suited, as it provides robust loadings and valid model fit information for models using binary indicators [52]. Measurement invariance (MI) across time and sex groups was tested. To assess the model fit, established fit indices were used with an excellent fit being indicated by a root mean square error of approximation (RMSEA) ≤ 0.05, a standardized root mean square residual (SRMR) ≤ 0.10, and a comparative fit index (CFI) ≥ 0.95 [53], whereby a CFI of > 0.90 is still considered acceptable [54]. 

**Fig. 1:**
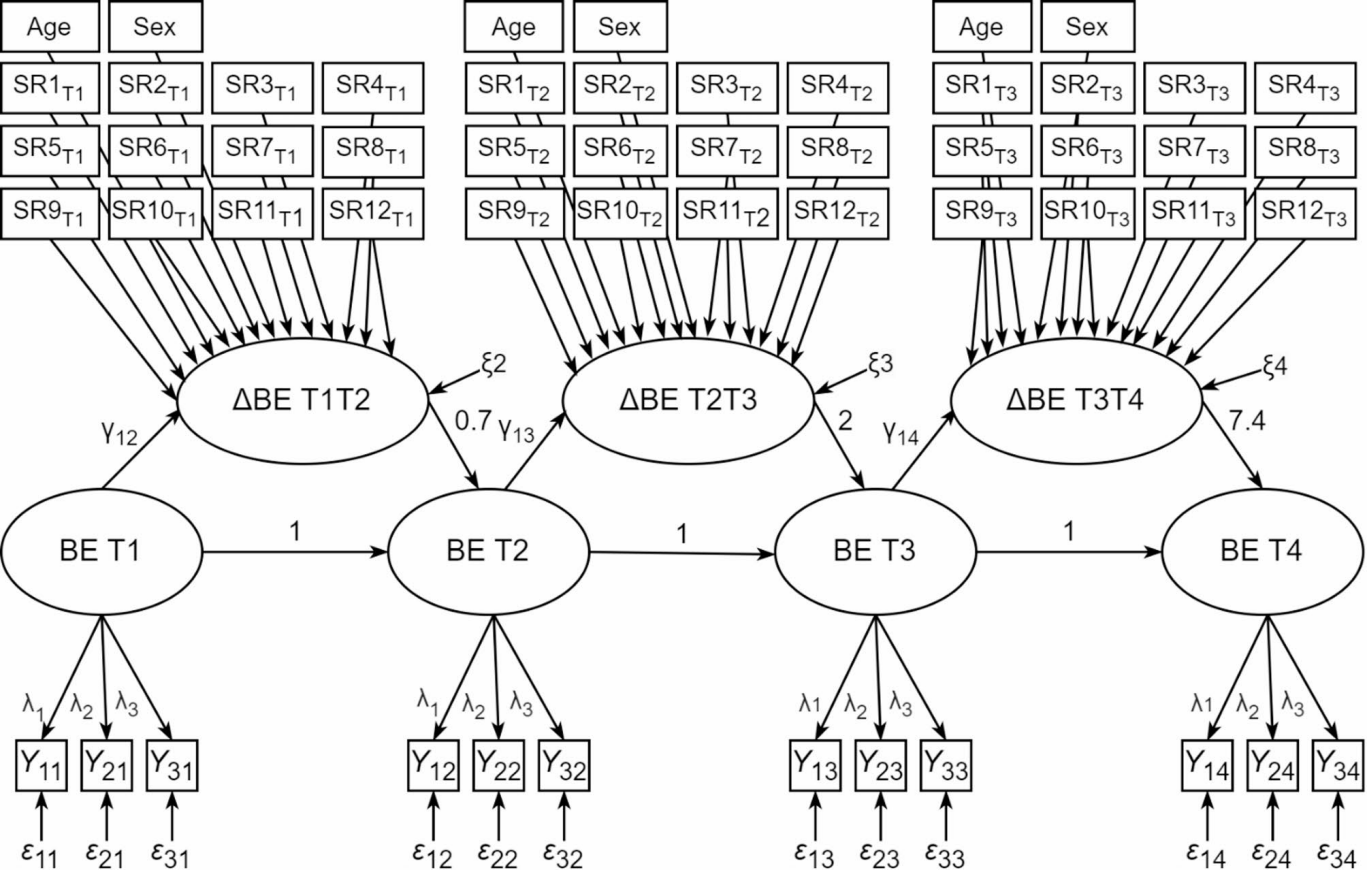
Illustration of the tested latent change score model. Intercorrelations between self-regulation facets are not depicted to increase clarity. SR = self-regulation, BE = binge eating, ΔBE T1T2 = latent change factor from T1 to T2; ΔBE T2T3 = latent change factor from T2 to T3; ΔBE T3T4 = latent change factor from T3 to T4; T1 = time point 1; T2 = time point 2; T3 = time point 3; T4 = time point 4

A multiple indicator latent change score model (LCSM) was employed to investigate the development of binge eating over time. LCSMs are a powerful tool for longitudinal investigations as they explicitly model true change as a latent variable, enabling the examination of both intra-individual change between time points and interindividual differences in changes [55]. Additionally, the use of multiple indicators allows to remove the measurement error and establish measurement invariance over time [55, 56]. As illustrated in Fig. 1, three latent change variables (ΔBE_T1T2_, ΔBE_T2T3_, ΔBE_T3T4_) were modelled to represent the changes in the true scores of binge eating between consecutive time points. Specifically, to model the latent change score ΔBE_T1T2_, the latent binge eating factor at T2 was regressed on the latent binge eating factor at T1 and a latent change factor fixed to 1 [57]. To account for unequal time intervals between measurement points, path coefficients between latent scores were scaled according to the time elapsed [58]. As a result, the estimated change parameters reflect changes projected to a one-year interval. The means and variances of the latent change score can be interpreted as average change in a one-year interval and individual variability in change, respectively. To investigate if self-regulation facets predict change in binge eating, we added the different self-regulation facets as predictors into the latent change score model. More specifically, self-regulation facets at T1 predicted the changes in binge eating from T1 to T2, at T2 from T2 to T3, and at T3 from T3 to T4. This approach allowed us to examine how individuals differ in their development of binge eating based on their prior levels of self-regulation. Correlations between self-regulation facets were allowed and age and sex were included as control variables. We performed two models, one with and one without controlling for BMI-SDS, to account for the high intercorrelation of binge eating and weight [13]. Additionally, multi-group analyses using model constraints were conducted to explore potential sex differences in the latent change scores, and their prediction through self-regulation. 

## Results

### Missing data

In total, 18.14% of data were missing. The percentage of missing values across the study variables and time points varied between 0% for sex and 65.7% for binge eating indicators at T4 [see Additional file 1, Table S1]. Given the high dropout rate at T4, we used binomial logistic regression to examine whether participation at T4 could be predicted by study variables from T1. The analysis revealed that participants who were younger at T1 (*b* = − 0.33, *p* <.001), with lower food responsiveness (*b* = − 0.20, *p* =.046), higher inhibitory control (*b* = 0.22, *p* =.048), and lower emotion regulation (*b* = − 0.27, *p* =.010) were less likely to participate at T4. Importantly, earlier binge eating did not predict participation at T4 (*b* = − 0.23, *p* =.500), we therefore assumed that T4 binge eating data were missing at random [59]. Thus, to account for missingness, we performed multiple imputations using chained equations with the *mice* package version 3.16.0 in R [60]. Multiple imputation produces reliable results and reduces bias under the missing at random assumption, and is widely recommended as the gold standard for handling missing data [61, 62], also when the proportion of missingness is large [63]. The existing data was used to generate *m* = 66 data sets of plausible values [64] with 50 iterations for each imputation using logistic regression for the binary binge eating indicators and predictive mean matching for all other variables. Convergence plots were checked and are presented in Additional file 2. To reduce a potential bias and increase efficiency [65], we included auxiliary variables [see Additional file 1, Table S2 ].

### Descriptive statistics and latent variable modelling of binge eating

As presented in Table 1, frequencies based on the non-imputed data ranged between 7.7% for overeating at T3 and 30.5% for loss of control eating at T4. Latent modeling was performed for the binge eating variable for the four time points, including three items each. Binge eating showed significant correlations across T1 to T3 (*r* ≥.55, *p* <.001), while binge eating at T4 was only significantly correlated with T3 (*r* =.21, *p* =.033) and not with earlier time points [see Additional file 1, Table S3]. Measurement invariance across time and sex was tested (Table 2). Given the binary binge eating indicators, the invariance of loadings and thresholds were tested simultaneously [66]. Scalar measurement invariance across time was established. This ensures that changes in the observed scores reflect true differences in the latent binge eating construct rather than measurement artifacts, thereby validating mean comparisons over time. This enabled us to examine both, the intraindividual change over time and the interindividual variability in change [55]. Full scalar measurement invariance across sex was narrowly missed, therefore the factor loading of one item (eating in the absence of hunger at T1) had to be freely estimated between groups. Partial scalar measurement invariance across sex was indicated.

**Table 1:** Frequencies and bivariate correlations for binge eating indicators

Indicator			Loss of control eating							Overeating							Eating in absence of hunger							
	TP	% Yes	T1				T2	T3	T4	T1				T2	T3	T4	T1					T2	T3	T4
Loss of control eating	T1	29.1	1																					
	T2	25.5	.24^***^				1																	
	T3	23.1	.20^***^				.23^***^	1																
	T4	30.5	.02				.03	.11^**^	1															
Overeating	T1	14.2	.12^***^				.06^*^	.09^***^	.01	1														
	T2	9.0	.08^**^				.12^***^	.05^*^	.01	.13^***^				1										
	T3	7.7	.07^**^				.06^*^	.10^***^	−.01	.06^*^				.14^***^	1									
	T4	26.7	−.01				.02	.02	.26^***^	.02				.09^*^	.02	1								
Eating in absence of hunger	T1	21.5	.18^***^				.15^***^	.13^***^	−.05	.14^***^				.16^***^	.10^***^	−.03	1							
	T2	16.1	.13^***^				.22^***^	.08^**^	−.07	.10^***^				.19^***^	.11^***^	.002	.22^***^					1		
	T3	14.1	.11^***^				.12^***^	.23^***^	.07	.09^**^				.10^***^	.14^***^	.12^***^	.17^***^					.20^***^	1	
	T4	21.4	.05				.05	.04	.36^***^	−.01				.04	−.02	.33^***^	.06					.03	.06	1

Note. TP = time point; T1 = time point 1; T2 = time point 2; T3 = time point 3; T4 = time point 4. *p <.05 **p <.01 ***p <.001

**Table 2:** Measurement invariance testing of binge eating

	χ^2^	df	RMSEA	ΔRMSEA	CFI	ΔCFI	SRMR	ΔSRMR
Across time								
Configural	115.95	30	0.041		0.955		0.049	
Scalar	132.77	41	0.036	0.005	0.952	0.003	0.052	0.003
Across sex								
Configural	161.54	64	0.042		0.949		0.059	
Partial Scalar	183.57	71	0.043	0.001	0.941	0.008	0.063	0.005
Scalar	192.37	72	0.045	0.003	0.936	0.013	0.064	0.006

Note. CFI = Comparative fit index; RMSEA = Root mean square error of approximation; SRMR = Standardized root mean square residual

### Latent change score model of binge eating

The latent change score model of binge eating showed a good model fit, equivalent to the scalar measurement invariance model of the state variables (χ^2^ = 132.77 (41), RMSEA = 0.036, CFI = 0.952, SRMR = 0.052). As illustrated in Table 3, across all participants an overall decrease in binge eating was found between T1 and T2 and no significant change between T2 and T3. An overall increase was observed between T3 and T4. Additionally, all variances of the change scores were significantly different from zero, indicating substantial significant interindividual differences in changes across development. Additionally, there was no significant correlation of the change score from T1-T2 with the initial state of binge eating (*r* = −.17, *p* =.224), showing that the initial level of binge eating was not associated with the following change [see Additional file 1, Table S4 for further correlations].

**Table 3:** Latent means and variances of binge eating

	µ	σ^2^
Binge eating T1	–0.57***	0.30***
Yearly change T1 to T2	–0.33***	0.46**
Yearly change T2 to T3	–0.03	0.09***
Yearly change T3 to T4	0.09***	0.019***

Note. µ = latent mean; σ^2^ = latent variance; ** *p* <.01 *** *p* <.001

### Prediction through self-regulation

**Fig. 2:**
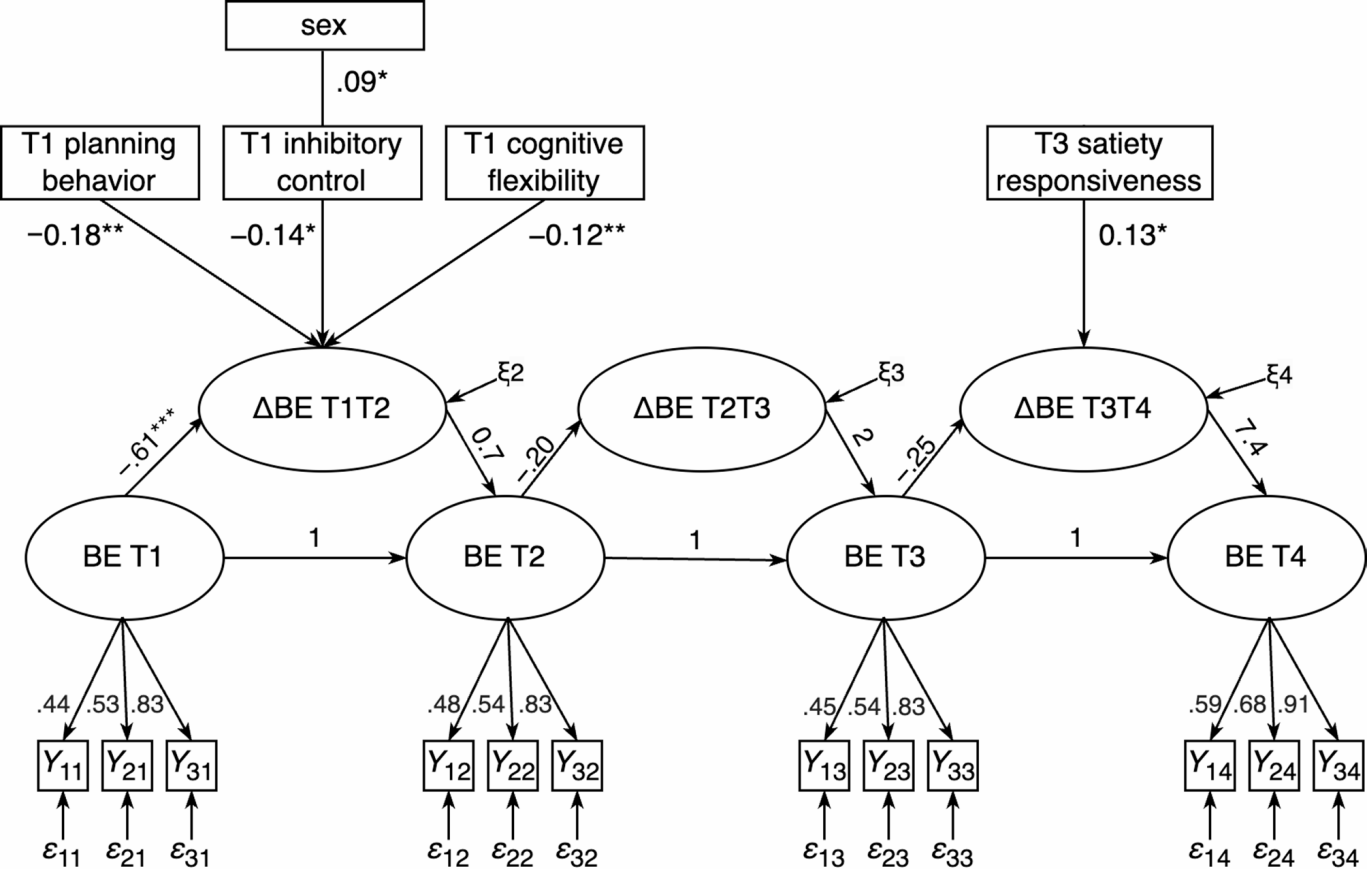
Significant predictions through self-regulation on binge eating development. Results of the latent change score model with only the significant paths being depicted. Sex defined as 1 = girls, 2 = boys. BE = binge eating, ΔBE T1T2 = latent change factor from T1 to T2; ΔBE T2T3 = latent change factor from T2 to T3; ΔBE T3T4 = latent change factor from T3 to T4; T1 = time point 1; T2 = time point 2; T3 = time point 3; T4 = time point 4

Next, we tested whether binge eating at T1 and changes in binge eating were predicted by the self-regulation facets. The model fit indicated an acceptable model fit (χ^2^ = 1706.14 (478), RMSEA = 0.039, CFI = 0.929, SRMR = 0.058). At T1, a higher occurrence of child-reported binge eating was cross-sectionally associated with lower planning behavior (β = −0.20, *p* <.001), lower inhibition (β = −0.13, *p* <.001), lower working memory updating (β = −0.17, *p* <.001), lower cognitive flexibility (β = −0.19, *p* <.001), lower inhibitory control (β = −0.16, *p* <.001), higher food responsiveness (β = 0.08, *p* =.038), higher external eating (β = 0.08, *p* =.039), male sex (β = 0.11, *p* =.011), and younger age (β = −0.09, *p* =.013). As presented in Fig. 2, a greater decrease in binge eating between T1 and T2 was predicted by higher T1 planning behavior (β = −0.18, *p* =.001), higher T1 inhibitory control (β = −0.14. *p* =.011), higher T1 cognitive flexibility (β = −0.12, *p* =.003), and female sex (β = 0.09, *p* =.021). No significant predictions through self-regulation facets at T2 on binge eating development T2-T3 were found (*p* ≥.121). A higher satiety responsiveness at T3 predicted a greater increase in binge eating between T3 and T4 (β = 0.13, *p* =.014). None of the other predictions through self-regulation facets at T3 reached significance. An additional model including the respective BMI-SDS at each time point as further control variable, yielded the same results regarding inter- and intraindividual changes in binge eating and the prediction through self-regulation.

### Sex differences

Multi-group analysis of sex for the latent change score model indicated an acceptable model fit (χ^2^ = 194.78 (77), RMSEA = 0.043, CFI = 0.938, SRMR = 0.063). As presented in Table 4, a significant sex difference was found for the initial level of binge eating, with male children reporting higher binge eating at T1 than female children. No significant sex differences emerged for the change scores (*p* ≥.380). To explore whether the prediction through self-regulation differs between boys and girls, a second multigroup latent change score model was conducted, incorporating self-regulation facets as predictors (χ^2^ = 1643.99 (869), RMSEA = 0.033, CFI = 0.950, SRMR = 0.045). No significant sex differences regarding the prediction through self-regulation were found (*p* ≥.100).

**Table 4:** Results of the multigroup analysis of the latent change score model by sex

	Female		Male		Difference	
	µ	σ^2^	µ	σ^2^	µ	σ^2^
Binge eating T1	−0.360	0.330***	−0.238	0.381***	0.122*	0.051
Yearly change T1 to T2	−0.315**	0.753**	−0.245**	0.843**	0.070	0.090
Yearly change T2 to T3	−0.010	0.071*	−0.048	0.096***	−0.038	0.025
Yearly change T3 to T4	0.093***	0.021***	0.079***	0.019***	−0.014	−0.002

Note. Non-standardized estimates. µ = latent mean; σ^2^ = latent variance; *p <.05 ** *p* <.01 *** *p* <.001

## Discussion

This prospective, community-based study investigated changes in binge eating over the course of middle childhood into adolescence. Results indicated a non-linear development of binge eating across childhood, first decreasing in early middle childhood then being stable and increasing across adolescence. Additionally, self-regulation was tested as a putative predictor for the development of binge eating. The simultaneous examination of multiple basal, complex, and appetite-related self-regulation facets enabled us to investigate the relative contribution of individual self-regulation facets and thereby identify particularly relevant facets The results showed that the self-regulation facets planning behavior, inhibitory control, and cognitive flexibility were particularly relevant, as they predicted the decrease in binge eating at the start of middle childhood. Contrary to theoretical expectations, higher satiety responsiveness predicted an increase in binge eating during adolescence. Controlling for BMI-SDS did not influence the results and multi-group analysis did not reveal any sex differences in the prediction models.

### Prevalence and course of binge eating

Regarding the indicators of binge eating, prevalences ranged between 8% and 31%, whereby loss of control eating was reported with the highest frequencies across middle childhood and adolescence, ranging between 23% and 31%. This prevalence is comparable to prior community-based studies [7, 8]. In general, the observed prevalence rates support the notion that binge eating manifests during middle childhood and adolescence. This is an important finding, particularly given the paucity of research on binge eating in this age group.

To further understand the development of binge eating, the course of binge eating was examined using a multiple indicator latent change score model. Concerning the intraindividual change, we observed a decrease in reported binge eating from T1 to T2. This aligns with previous studies demonstrating spontaneous remission of loss of control eating and overeating during middle childhood in up to half of all children [67, 68]. This phenomenon could provide an explanation for the overall reduction of binge eating between T1 and T2. The observed increase during adolescence (T3 to T4) aligns with our expectations and earlier research [67, 69], indicating adolescence as a critical period for the prevention of binge eating. Furthermore, the latent change score model revealed significant true variances of the change score factors throughout development, suggesting substantial interindividual differences in the course of binge eating, e.g., regarding the direction or the magnitude of change. Given the observed intra- and interindividual differences, our results underpin that the period of childhood and adolescence is a crucial developmental stage in the prevention of binge eating [11].

### Prediction of binge eating through self-regulation

The interindividual differences in binge eating suggest the presence of intraindividual factors that influence binge eating emergence and development. Previous research, predominantly cross-sectional, has highlighted the importance of self-regulation [15]. Therefore, we investigated whether different facets of self-regulation could predict subsequent changes in binge eating. Furthermore, simultaneously including multiple self-regulation facets allowed us to examine which self-regulation facets indicate the highest impact on binge eating. At T1, higher binge-eating was cross-sectionally associated with lower planning behavior, lower inhibition, lower working memory updating, lower inhibitory control, lower cognitive flexibility, higher food responsiveness, and higher external eating. These results are consistent with research indicating cross-sectional associations during childhood between loss of control eating in children with overweight and planning behavior [70], loss of control eating and inhibition for a systematic review see [31], disinhibited eating and working memory for a meta-analysis see [71], loss of control eating as well as eating in the absence of hunger and cognitive flexibility [35], and binge eating and external eating [72]. These findings suggest an association between various facets of self-regulation and binge eating during middle childhood. However, these results are cross-sectional and do not provide evidence for the direction of effects, or the influence of self-regulation on changes over time [32].

To overcome these limitations, we further examined whether self-regulation precedes the development of binge eating. Results indicated that a decrease in binge eating between T1 and T2 was significantly predicted by higher levels of planning behavior, inhibitory control, and cognitive flexibility. A recent longitudinal study by Davis et al. (2019) indicated that lower planning behavior at the mean age of 12.8 predicted higher binge eating three years later [34]. Although their results refer to a different age group, they underline the potential importance of planning behavior in preventing binge eating. Children who plan well and successfully pursue a goal might be less prone to developing binge eating symptoms. Additionally, planning behavior is discussed to bridge the gap between intentions and actual behavior, and is therefore an important starting point for health interventions [73]. Regarding cognitive flexibility, our results propose that children who are better at switching between tasks and are more flexible to alternative actions show a subsequent decrease in binge eating. Cognitive flexibility could therefore be an effective protective factor, as switching between different cognitions might support the transition from maladaptive to adaptive eating habits as it enables children to abandon suboptimal approaches (e.g., continue eating) in favour of more effective methods to achieve a certain goal [25]. Contrary to this idea and our findings, Shields et al. [71] found in their systematic review and meta-analysis no association between cognitive flexibility and disinhibited eating. However, only three cross-sectional studies were included, highlighting the need for further investigations. Lastly, inhibitory control emerged as a significant predictor of a reduction in binge eating. It seems reasonable that children who find it easier to suppress inappropriate impulses (e.g., to eat more) are less likely to engage in binge eating. In summary, our results suggest that planning behavior, inhibitory control, and cognitive flexibility may function as protective factors against binge eating during middle childhood. These findings support the notion that binge eating can be seen as a reaction to stressful events, in which higher self-regulation helps to show adaptive reactions other than binge eating [26]. Therefore, one potential starting point for prevention and intervention programs regarding binge eating in middle childhood could be self-regulation. Future studies could build on these findings by applying person-centred approaches (e.g., latent class analysis and growth mixture modelling) to analyse individual binge eating trajectories and examine the extent to which these are predicted by self-regulation abilities.

Contrary to our hypothesis that the strongest predictions through self-regulation were expected during adolescence (T3-T4), only higher satiety responsiveness at T3 predicted a higher increase in binge eating during adolescence. A potential explanation for this finding may be, that children with higher satiety responsiveness may also feel more likely to lose control over their eating due to their greater interoceptive ability. As we only investigated subjective binge eating, we do not know whether the feeling of loss of control was accompanied by eating an objectively large amount of food. Interestingly, Poovey and Rancourt [74] found that binge eating was associated with higher visceral sensitivity (e.g., heightened sensitivity to gastrointestinal signals) in an undergraduate sample. Furthermore, they found that higher satiety responsiveness predicted the membership in a disordered eating profile including binge eating, but no significant prediction emerged for a profile consisting primarily of binge eating. The same is true for a longitudinal study where no association between satiety responsiveness at the age of 4 to 5 and binge eating at the age of 12 to 14 was observed [75]. Research on the relationship between satiety responsiveness and binge eating during middle childhood and adolescence remains limited and given the literature on an association between lower interoception and disordered eating [76], the present pathway must be interpreted with caution and requires further replication.

No significant predictions emerged for emotional reactivity, working memory updating, inhibition, affective decision-making, anger regulation, emotional overeating, food responsiveness, and external eating. These non-significant findings contrast with our initial hypotheses. While both earlier studies [31, 71, 72] and our own results identified several significant cross-sectional associations, they might not persist in a longitudinal context. This highlights the need for future research to examine prospective data bidirectionally and investigate whether previous cross-sectional findings may reflect a reverse temporal order with binge eating leading to reduced self-regulation [34]. Furthermore, our data are based on a community sample and different patterns might emerge in clinical samples with a higher prevalence of binge eating. Supporting this, a meta-analysis in adults demonstrated that differences in self-regulation are evident in clinical binge eating disorder, but not in non-clinical binge eating [3]. Additionally, since self-regulation undergoes significant developmental changes during childhood and adolescence, the relatively long intervals of approximately 2 years between T2 and T3 and 8 years between T3 and T4 may have been too long to detect significant predictions. Future research should investigate the interplay of binge eating and self-regulation in a narrower time frame e.g., through ecological momentary assessment [77], to gain deeper insights into their dynamic interplay on a daily basis.

Concerning sex differences, boys reported significantly more binge eating compared to girls at the start of the study (T1). No further sex differences for the course of binge eating were found, which is consistent with prior research [11, 69, 78]. Furthermore, in line with prior cross-sectional analyses [71], no significant sex differences were found regarding the prediction of binge eating through self-regulation. Previously, weight status was frequently highlighted as an influential factor in the development of binge eating [13]. Upon controlling the tested model for BMI-SDS, variance in the latent change scores remained significant, suggesting that the variance between children cannot be attributed exclusively to the weight status, which aligns with previous research [79]. The observed predictions of self-regulation facets on changes in binge eating were also found to be independent of body weight.

### Strengths and limitations

The results of our prospective community-based study must be considered in the context of strengths and limitations. First, using the preregistered and powerful approach of a latent change score model allowed us to investigate the development of binge eating over ten years. Additionally, different indicators were used for the latent modelling of binge eating, thereby minimizing the measurement error. However, only child-report measures of binge eating were incorporated. Including multiple sources [80] or a clinical interview [69] would have enhanced the reliability of our results. Second, several self-regulation facets could be examined simultaneously, allowing us to analyze their unique contribution in the prediction of changes in binge eating. As the data on self-regulation facets stem from different informants (child-report, parent-report, teacher-report), as well as measures (questionnaires, experimental data), biases due to single measures were reduced. While reliability for most of the questionnaires was high, others indicated lower reliabilities (e.g., anger regulation at T4). This may have been caused by the use of shortened scales due to time constraints [19] and has to be considered when interpreting the respective results, as the likelihood for Type II errors may be enhanced. Lastly, although missing data is common in longitudinal studies, and our overall rate of missing data falls below the average of 26.5% [81], we must specifically address the high dropout between T3 and T4. To account for missing data, we performed the multiple imputation approach that has been found to produce reliable results [61].

## Conclusion

Our results highlight childhood and adolescence as a critical period for the development of binge eating, as intra- and interindividual differences in the development of binge eating were observed. More specifically, during early middle childhood, a decline in binge eating was identified. Subsequent to a period of stability, adolescence was marked by a rise in binge eating. Differences between participants in binge eating emerged throughout the study period. The simultaneous examination of multiple self-regulation facets revealed that cognitive flexibility, inhibitory control, and planning behavior may serve as protective self-regulation facets during middle childhood. In contrast to our expectations, higher satiety responsiveness emerged as a risk factor for binge eating during adolescence. Our findings underscore the importance of middle childhood as an early period for prevention and intervention strategies. Strengthening specific self-regulation facets may offer a valuable approach for such prevention and intervention strategies. Future research should investigate potential prospective and bidirectional associations between self-regulation and binge eating, while considering shorter intervals between assessments. 

## Electronic supplementary material

Below is the link to the electronic supplementary material.

Supplementary Material 1

Supplementary Material 2

## Data Availability

The datasets generated and analyzed during the current study are not publicly available, as the participants were not asked to consent to publication within repositories but are available from the corresponding author upon reasonable request.

## Abbreviations

BMI-SDS: Standard deviation score of the body mass index
BRIEF: Behavior rating inventory of executive function
CFI: Comparative fit index
LCSM: Latent change score model
MI: Measurement invariance
RMSEA: Root mean square error of approximation
SRMR: Standardized root mean square residual
T1: Time point 1
T2: Time point 2
T3: Time point 3
T4: Time point 4
WLSMV: Weighted least squares mean and variance-adjusted
