# Supplementary material for: From childhood to adolescence: Development of binge eating and the prospective role of self-regulation
Source: J Eat Disord. 2025 Jul 6;13:129. doi: 10.1186/s40337-025-01330-x (PMC12232867; doi:10.1186/s40337-025-01330-x)
Supplement: Supplementary file 1 — Supplementary Material 1 [file 40337_2025_1330_MOESM1_ESM.docx]

**ADDITIONAL FILE I**

From Childhood to Adolescence: Development of Binge Eating and the Prospective Role of Self-Regulation

**Authors**

Nele Westermann^1^: [nele.westermann.1@uni-potsdam.de](mailto:nele.westermann.1@uni-potsdam.de), [ORCID: 0000-0002-4791-2336](https://orcid.org/0000-0002-4791-2336)

Annette M. Klein^2^: [annette.klein@ipu-berlin.de](mailto:annette.klein@ipu-berlin.de), [ORCID: 0000-0001-8246-4666](https://orcid.org/0000-0001-8246-4666)

Robert Busching^1^: busching@uni-potsdam.de, [ORCID: 0000-0001-7522-6053](https://orcid.org/0000-0001-7522-6053)

Petra Warschburger^1^ (correspondence author): [warschb@uni-potsdam.de](mailto:warschb@uni-potsdam.de), [ORCID: 0000-0001-7979-7451](https://orcid.org/0000-0001-7979-7451)

^1^University of Potsdam, Department of Psychology, Karl-Liebknecht-Straße 24/25, 14476 Potsdam, Germany

^2^International Psychoanalytic University Berlin, Stromstr. 1, 10555 Berlin, Germany

**Table S1.**

*Percentage of missing values per variable and time point*

| Variable | missing T1  in % | missing T2  in % | missing T3  in % | missing T4  in % |
| --- | --- | --- | --- | --- |
| Binge eating | 0.96 | 4.10 | 9.46 | 65.72 |
| Emotional reactivity | 19.40 | 28.07 | 35.84 | – |
| Working-memory updating | 1.14 | 3.19 | 9.88 | – |
| Cognitive flexibility | 1.14 | 4.46 | 10.78 | – |
| Inhibition | 0.96 | 3.31 | 10.24 | – |
| Inhibitory control | 19.58 | 18.25 | 35.84 | – |
| Planning behavior | 14.52 | 30.00 | 33.25 | – |
| Affective decision-making | 1.02 | 4.28 | 10.78 | – |
| Satiety responsiveness | 19.46 | 28.19 | 35.96 | – |
| Emotional overeating | 19.82 | 28.49 | 36.27 | – |
| Food responsiveness | 19.46 | 28.13 | 35.96 | – |
| External eating | 19.52 | 28.31 | 36.14 | – |
| Anger regulation | 19.46 | 28.19 | 35.96 | – |
| BMI-SDS | 1.02 | 3.25 | 11.57 | – |
| Sex | 0.00 | – | – | – |
| Age | 0.18 | – | – | – |

*Note.* T1 = time point 1, T2 = time point 2, T3 = time point 3, T4 = time point 4.

**Table S2.**

*List of auxiliary variables used in multiple imputation*

| Auxillary variable | Name in plots | Time point(s) | Source |
| --- | --- | --- | --- |
| Language spoken at home | AVMig | T1 | Parent |
| Highest parental education level | AVBild | T1, T2 | Parent |
| Household income | AVEINK | T2, T3 | Parent |
| Familial risk factors | AVFR | T2, T3 | Parent |
| Achievement motivation in school | AVLeiM | T3 | Child |
| Self-esteem | AVSW | T1, T2, T3 | Child |
| Body dissatisfaction | AVKUm | T1, T2, T3 | Child |
| Unhealthy snacking behavior | AVSnack | T3 | Child |
| Weight stigma | AVWS | T1, T2, T3 | Child |
| Internalization of weight stigma | AVWBIS | T3 | Child |
| Emotional and behavioral problems | AVSDQ | T1, T2, T3 | Parent |
| Delay of gratification | AVDOG | T1, T2, T3 | Child |
| Need for cognition | AVNFC | T3 | Child |
| Grade in reading | AVNotL | T1, T2, T3 | Parent |
| Grade in spelling | AVNotR | T1, T2, T3 | Parent |
| Grade in mathematics | AVNotM | T3 | Parent |
| Academic performance | AVPLF | T1, T2, T3 | Teacher |
| Processing speed | AVZST | T1, T2, T3 | Child |

*Note.* T1 = time point 1, T2 = time point 2, T3 = time point 3.

**Table S3.**

*Descriptive statistics and bivariate correlations of study variables*

| Variable (potential range) | *M* (*SD*) | Imputed  *M* (*SD*) | Bivariate correlations | | | |
| --- | --- | --- | --- | --- | --- | --- |
|  |  |  | T1 BE | T2 BE | T3 BE | T4 BE |
| T1 BE (0-1) | 0.17 (0.20) | 0.22 (0.27) | – |  |  |  |
| T2 BE (0-1) | 0.13 (0.19) | 0.17 (0.25) | .74*** | – |  |  |
| T3 BE (0-1) | 0.13 (0.18) | 0.16 (0.24) | .61*** | .55*** | – |  |
| T4 BE (0-1) | 0.23 (0.26) | 0.42 (0.37) | .08 | .10 | .21* | – |
| T1 Cognitive flexibility (0-22) | 15.39 (4.78) | 15.38 (4.78) | −.24*** | −.21*** | −.22*** | −.11 |
| T2 Cognitive flexibility (0-22) | 18.14 (3.92) | 18.10 (3.95) | −.25*** | −.21*** | −.23*** | −.15** |
| T3 Cognitive flexibility (0-12) | 9.77 (1.87) | 9.74 (1.91) | −.03 | −.08*** | −.07 | −.07 |
| T1 Updating (0-13) | 6.18 (1.47) | 6.18 (1.47) | −.22*** | −.11** | −.11* | −.04 |
| T2 Updating (0-13) | 6.63 (1.51) | 6.62 (1.51) | −.19*** | −.19*** | −.17*** | −.07 |
| T3 Updating (0-13) | 7.38 (1.63) | 7.36 (1.64) | −.15*** | −.14*** | −.13** | −.10 |
| T1 Inhibition (n.a.) | −24.95 (8.78) | −24.94 (8.80) | −.18*** | −.16 ** | .14** | −.04 |
| T2 Inhibition (n.a.) | −20.55 (6.90) | −20.65 (6.98) | −.20*** | −.16*** | −.19*** | −.04 |
| T3 Inhibition (n.a.) | −16.72 (5.31) | −16.74 (5.33) | −.14** | −.12 ** | −.09* | −.02 |
| T1 Emotional reactivity (1-5) | 2.21 (0.71) | 2.22 (0.72) | .09 | .10* | .16** | .06 |
| T2 Emotional reactivity (1-5) | 2.14 (0.68) | 2.17 (0.70) | .05 | .08 | .16*** | .03 |
| T3 Emotional reactivity (1-5) | 2.25 (0.73) | 2.28 (0.76) | .06 | .17*** | .19*** | −.06 |
| T1 Emotional DM (−50-50) | 5.49 (11.43) | 5.48 (11.43) | −.05 | .02 | −.08 | −.09 |
| T2 Emotional DM (−50-50) | 8.45 (12.94) | 8.40 (12.93) | −.06 | −.10* | −.07 | −.02 |
| T3 Emotional DM (−50-50 | 9.56 (13.69) | 9.53 (13.69) | −.08 | −.10* | −.10* | −.08 |
| T1 Anger regulation (1-3) | 2.18 (0.66) | 2.19 (0.67) | −.01 | −.04 | .02 | .08 |
| T2 Anger regulation (1-3) | 2.24 (0.63) | 2.26 (0.65) | .03 | −.01 | −.02 | .14** |
| T3 Anger regulation (1-3) | 2.32 (0.67) | 2.34 (0.68) | .03 | −.04 | −.09 | .08 |
| T1 Inhibitory control (1-5) | 3.53 (0.67) | 3.51 (0.68) | −.21*** | −.22*** | −.27*** | −.10 |
| T2 Inhibitory control (1-5) | 3.59 (0.63) | 3.55 (0.65) | −.11* | −.21*** | −.23*** | −.13*** |
| T3 Inhibitory control (1-5) | 3.75 (0.68) | 3.70 (0.70) | −.16** | −.24*** | −.26*** | −.13*** |
| T1 Planning behavior (1-5) | 3.70 (0.89) | 3.71 (0.89) | −.28*** | −.23*** | −.31*** | −.22*** |
| T2 Planning behavior (1-5) | 3.65 (0.90) | 3.67 (0.90) | −.29*** | −.21*** | −.31*** | −.24*** |
| T3 Planning behavior (1-5) | 3.67 (0.96) | 3.67 (0.96) | −.26*** | −.11*** | −.30*** | −.17** |
| T1 Satiety respons (1-5) | 2.83 (0.77) | 2.84 (0.78) | −.05 | −.02 | −.05 | .05 |
| T2 Satiety respons (1-5) | 2.78 (0.77) | 2.79 (0.78) | −.04 | −.08 | −.03 | .10 |
| T3 Satiety respons (1-5) | 2.63 (0.77) | 2.64 (0.78) | −.08 | −.05 | −.01 | .08 |
| T1 Food respons (1-5) | 1.61 (0.89) | 1.63 (0.90) | .11* | .11* | .18*** | .14* |
| T2 Food respons (1-5) | 1.61 (0.90) | 1.66 (0.94) | .13** | .17*** | .21*** | .12* |
| T3 Food respons (1-5) | 1.62 (0.92) | 1.70 (0.99) | .12** | .12** | .23*** | .18** |
| T1 Emotional overeating (1-5) | 1.22 (0.41) | 1.23 (0.43) | −.02 | .07 | .11* | .19*** |
| T2 Emotional overeating (1-5) | 1.22 (0.39) | 1.24 (0.42) | −.003 | .06 | .10* | .07 |
| T3 Emotional overeating (1-5) | 1.27 (0.47) | 1.31 (0.52) | .01 | .04 | .18*** | .16** |
| T1 External eating (1-4) | 2.90 (0.57) | 2.90 (0.57) | .11* | .06 | .14* | .05 |
| T2 External eating (1-4) | 2.90 (0.58) | 2.91 (0.59) | .09* | .14** | .12* | .08 |
| T3 External eating (1-4) | 2.91 (0.47) | 2.92 (0.58) | .07 | .06 | .14* | .10 |
| T1 Age (n.a.) | 8.36 (0.95) | 8.36 (0.95) | −.10** | −.08** | −.01 | .06 |
| T2 Age (n.a.) | 9.11 (0.93) | (n.a.) | −.08** | −.05* | −.004 | −.03 |
| T3 Age (n.a.) | 11.06 (0.92) | (n.a.) | −.07** | −.06* | .01 | −.03 |
| T4 Age (n.a.) | 18.92 (1.04) | (n.a.) | −.06 | −.02 | .04 | .02 |
| T1 Sex (n.a.) | n.a. | n.a. | .12* | .14** | .07 | −.01 |
| T1 BMI-SDS (n.a.) | 0.16 (0.99) | 0.16 (0.99) | .09* | .08 | .08 | .08 |
| T2 BMI-SDS (n.a.) | 0.20 (0.99) | 0.20 (0.99) | .08* | .09* | .07 | .07 |
| T3 BMI-SDS (n.a.) | 0.24 (1.07) | 0.24 (1.07) | .08* | .11** | .13** | .11* |

*Note.* T1 = time point 1; T2 = time point 2; T3 = time point 3; T4 = time point 4; BE = binge eating*;* DM = decision making; Responsiv = responsiveness; BMI-SDS = standardized body mass index; n.a. = not applicable.

**p* < .05 ** *p* < .01 *** *p* <.001

**Table S4**

*Latent means, variances, and bivariate correlations of binge eating*

| Latent variable | μ | σ^2^ | Bivariate correlations | | |
| --- | --- | --- | --- | --- | --- |
|  |  |  | 1 | 2 | 3 |
| 1 Binge eating T1 | –0.57*** | 0.30*** |  |  |  |
| 2 Yearly change T1 to T2 | –0.33*** | 0.46** | −.17 |  |  |
| 3 Yearly change T2 to T3 | –0.03 | 0.09*** | −.13 | −.55*** |  |
| 4 Yearly change T3 to T4 | 0.09*** | 0.019*** | −.31** | .00 | .20 |

*Note.* μ = latent mean; σ^2^ = latent variance; T1 = time point 1; T2 = time point 2; T3 = time point 3; T4 = time point 4; ** *p* < .01 *** *p* <.001.

**Table S5**

*Deviations from preregistration*

| **Deviation** | **Reason** |
| --- | --- |
| Use of multiple imputation instead of FIML to account for missing data | In deviation from preregistration, we performed multiple imputations to account for missing data. The WLSMV estimator did not allow the usage of full information maximum likelihood (FIML) which we were unaware of at the time of preregistration. |
| Naming of the variables: Food responsiveness and satiety responsiveness | In the methods section of the preregistration, we accidentally named the facets food responsiveness food responsibility, and satiety responsiveness satiety responsibility. This was of course changed in the manuscript. |
| Controlling for age | We originally only specified controlling our model by sex. However, given the dynamic development of self-regulation during middle childhood and adolescence we additionally controlled for age. |

*Note.* Preregistration is available at <https://osf.io/jaq4h>
