# Supplementary material for: From childhood to adolescence: Development of binge eating and the prospective role of self-regulation
Source: J Eat Disord. 2025 Jul 6;13:129. doi: 10.1186/s40337-025-01330-x (PMC12232867; doi:10.1186/s40337-025-01330-x)
Supplement: Supplementary file 2 — Supplementary Material 2 [file 40337_2025_1330_MOESM2_ESM.docx]

**ADDITIONAL FILE II – CONVERGENCE PLOTS**

From Childhood to Adolescence: Development of Binge Eating and the Prospective Role of Self-Regulation

**Authors**

Nele Westermann^1^: [nele.westermann.1@uni-potsdam.de](mailto:nele.westermann.1@uni-potsdam.de), [ORCID: 0000-0002-4791-2336](https://orcid.org/0000-0002-4791-2336)

Annette M. Klein^2^: [annette.klein@ipu-berlin.de](mailto:annette.klein@ipu-berlin.de), [ORCID: 0000-0001-8246-4666](https://orcid.org/0000-0001-8246-4666)

Robert Busching^1^: busching@uni-potsdam.de, [ORCID: 0000-0001-7522-6053](https://orcid.org/0000-0001-7522-6053)

Petra Warschburger^1^ (correspondence author): [warschb@uni-potsdam.de](mailto:warschb@uni-potsdam.de), [ORCID: 0000-0001-7979-7451](https://orcid.org/0000-0001-7979-7451)

^1^University of Potsdam, Department of Psychology, Karl-Liebknecht-Straße 24/25, 14476 Potsdam, Germany

^2^International Psychoanalytic University Berlin, Stromstr. 1, 10555 Berlin, Germany

**Multiple imputation convergence plots**
